# Effects of virtual reality-based rehabilitation on distal upper extremity function and health-related quality of life: a single-blinded, randomized controlled trial

**DOI:** 10.1186/s12984-016-0125-x

**Published:** 2016-02-24

**Authors:** Joon-Ho Shin, Mi-Young Kim, Ji-Yeong Lee, Yu-Jin Jeon, Suyoung Kim, Soobin Lee, Beomjoo Seo, Younggeun Choi

**Affiliations:** National Rehabilitation Center, Ministry of Health and Welfare, Seoul, Korea; Department of Law, Hanyang University, Seoul, Korea; Neofect, Yong-in, Korea; School of Games, Hongik University, Seoul, Korea; Department of Applied Computer Engineering, Dankook University, Yong-in, Korea; Department of Rehabilitation Medicine, National Rehabilitation Center, Ministry of Health and Welfare, Samgaksan-ro 58, Gangbuk-gu, Seoul 142-884 Korea

**Keywords:** Rehabilitation, Upper extremity, Virtual reality therapy, Quality of life, Video games

## Abstract

**Background:**

Virtual reality (VR)-based rehabilitation has been reported to have beneficial effects on upper extremity function in stroke survivors; however, there is limited information about its effects on distal upper extremity function and health-related quality of life (HRQoL). The purpose of the present study was to examine the effects of VR-based rehabilitation combined with standard occupational therapy on distal upper extremity function and HRQoL, and compare the findings to those of amount-matched conventional rehabilitation in stroke survivors.

**Methods:**

The present study was a single-blinded, randomized controlled trial. The study included 46 stroke survivors who were randomized to a Smart Glove (SG) group or a conventional intervention (CON) group. In both groups, the interventions were targeted to the distal upper extremity and standard occupational therapy was administered. The primary outcome was the change in the Fugl–Meyer assessment (FM) scores, and the secondary outcomes were the changes in the Jebsen–Taylor hand function test (JTT), Purdue pegboard test, and Stroke Impact Scale (SIS) version 3.0 scores. The outcomes were assessed before the intervention, in the middle of the intervention, immediately after the intervention, and 1 month after the intervention.

**Results:**

The improvements in the FM (FM-total, FM-prox, and FM-dist), JTT (JTT-total and JTT-gross), and SIS (composite and overall SIS, SIS-social participation, and SIS-mobility) scores were significantly greater in the SG group than in the CON group.

**Conclusions:**

VR-based rehabilitation combined with standard occupational therapy might be more effective than amount-matched conventional rehabilitation for improving distal upper extremity function and HRQoL.

**Trial registration:**

This study is registered under the title “Effects of Novel Game Rehabilitation System on Upper Extremity Function of Patients With Stroke” and can be located in https://clinicaltrials.gov with the study identifier NCT02029651.

## Background

Regaining upper extremity function is one of the major goals in stroke survivors, as it is important for performing activities of daily living (ADLs). However, approximately 80 % of stroke survivors have upper extremity limitations, and these limitations persist in approximately half of these survivors in the chronic phase [1, 2]. Distal upper extremity function is vital for performing ADLs, such as holding objects like utensils, turning a doorknob or key in a lock, telephone or computer use, and writing, and is strongly related to quality of life (QoL) in stroke survivors [3]. In stroke survivors, the distal upper extremity is severely affected and is the last body part to recover [4]. Therefore, improving distal upper extremity function is of primary importance in the rehabilitation of stroke survivors.

Recent studies have emphasized the use of interventions that are focused and repetitive, relevant to real-life, and actively performed in order to promote cortical reorganization and neuroplasticity [5–8]. In this context, conventional interventions have been complemented by novel technologies such as virtual reality (VR).

VR-based rehabilitation is promising in stroke survivors, and many types of VR-based rehabilitation apparatus from commercial video game equipment to robotics are currently being developed and used. In the area of upper limb rehabilitation, a large number of studies have been performed in stroke survivors, and a recent systematic review concluded that the use of VR-based rehabilitation is superior to amount-matched conventional rehabilitation for improving upper limb function [9]. Nevertheless, most studies on VR-based rehabilitation for the upper extremity reported on the proximal upper extremity, with limited information on the distal upper extremity. Although 2 previous studies showed promising results regarding VR-based rehabilitation for the distal upper extremity, these studies did not include a control group [10, 11]. Randomized control trials have been performed using a VR system with different types of gloves; however, a definite conclusion about the treatment effect could not be obtained owing to the low number of participants [12, 13]. Furthermore, the effects of VR-based rehabilitation on health related quality of life (HRQoL) have not been appropriately assessed, although the QoL of stroke survivors is crucial for comprehensive rehabilitation.

Therefore, the objective of the present study was to examine the effects of VR-based rehabilitation combined with standard occupational therapy (OT) on distal upper extremity function and HRQoL, and compare the findings to those of amount-matched conventional rehabilitation in stroke survivors.

## Methods

### Study design

The present study was a single-blinded, randomized controlled trial performed at National Rehabilitation Center, an urban rehabilitation hospital in Seoul, Korea. Eligible participants were randomly assigned to a Smart Glove (SG) or conventional intervention (CON) group using a computer-generated randomized scheme. The allocation was performed using sealed opaque envelopes with the group name, which were placed in a plastic container in numerical order. Randomization, outcome measurements, and data analysis were performed by different individuals who were not involved in the intervention. This study was registered at clinicaltrials.gov (NCT02029651) and was approved by the Ethics Committee of the National Rehabilitation Center, Korea. All participants provided informed written consent before enrollment.

### Participants

The study included 46 consecutive participants with upper extremity functional deficits caused by stroke, who were present in a rehabilitation hospital. The inclusion criteria were as follows: (1) first-ever ischemic or hemorrhagic stroke; (2) complaints of unilateral upper extremity functional deficits after stroke; and (3) presence of a score of at least 2 points on the medical research council scale [14] for wrist flexion/extension or forearm pronation/supination, as the SG system can be operated only with volitional movements and does not involve external assistance. The exclusion criteria were as follows: (1) age <18 years; (2) uncontrolled hypertension, unstable angina, recent myocardial infarction, or any history of seizure; (3) predisposing psychological disorders that could impede participation; (4) neurological disorders that cause motor deficits, such as Parkinson’s disease and peripheral neuropathy; (5) severe aphasia resulting in communication difficulties that could influence the intervention and outcome measures; (6) cognitive impairment resulting in cooperation difficulties (a score of ≤24 in the Mini-Mental State Examination) [15]; and (7) severe pain impeding upper extremity rehabilitation (numeric pain rating scale score ≥ 7) [16].

### Intervention

All participants received a 4-week face-to-face intervention program (SG or CON) individually (20 sessions for 30 min per day) in a room for the intervention, as well as standard OT daily for 30 min in a room for OT. The intervention programs exclusively focused on the distal upper extremity and were administered by 3 trained occupational therapists who were involved in both the interventions and were exclusively dedicated to this study. The therapists were sequentially allocated such that the intervention to be performed by each therapist was automatically selected based on the randomized allocation of the SG and CON groups in order to minimize therapist bias. The intervention time was the same in both the groups. Standard OT involved range of motion and strengthening exercises for the affected limb, table-top activities, and training for ADLs and was administered by occupational therapists who were not involved in this study.

### Smart glove intervention

**Fig. 1:**
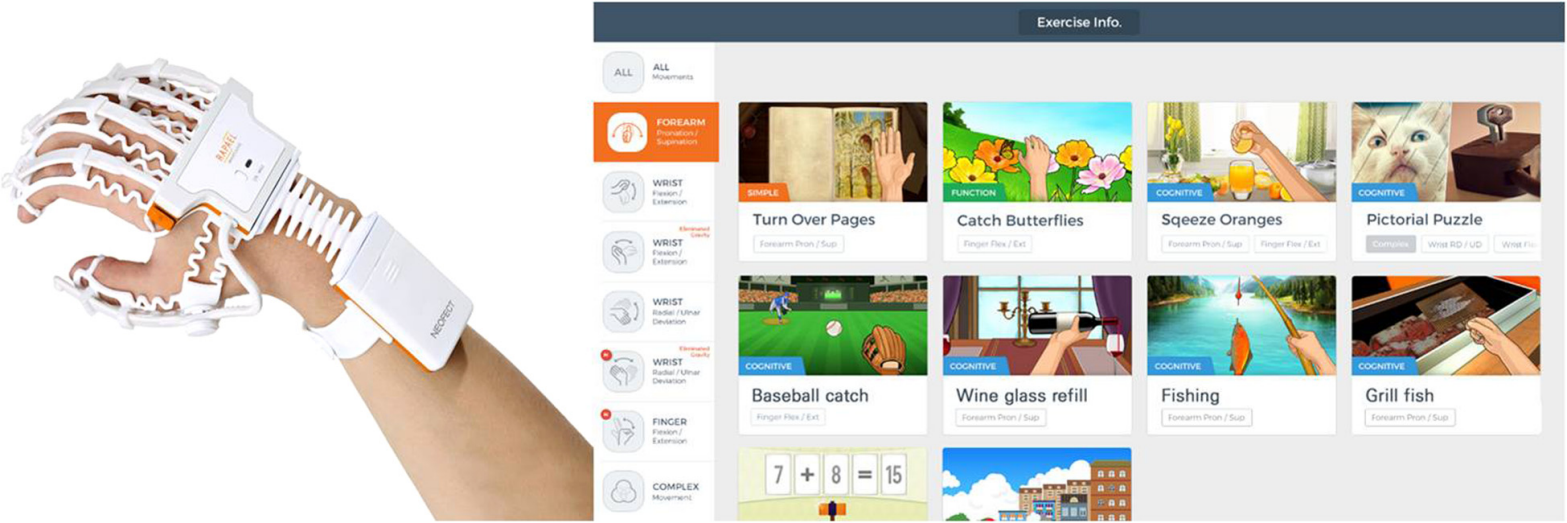
The RAPAEL Smart Glove™ system and the task-specific games of this system

The RAPAEL Smart Glove™ (Neofect, Yong-in, Korea) is a biofeedback system designed for distal upper extremity rehabilitation in stroke survivors (Fig. 1). It includes a glove-shaped sensor device and a software application. The sensor device tracks the motion and posture of the wearer’s distal limb and recognizes functional movements, such as forearm pronation/supination, wrist flexion/extension, radial-ulnar deviation, and finger flexion/extension. An inertial measurement unit sensor in the device measures the 3-dimensional orientation of the distal limb, and 5 bending sensors estimate the degree of bending of the fingers. The gathered sensing data is transmitted and received via wireless communication systems such as Bluetooth. The software application manipulates virtual hands or virtual objects in training games according to the received data. In addition, this system can evaluate the active and passive range of motion for each functional movement.

The training games in the SG system are categorized according to the intended movements as follows: forearm pronation/supination, wrist flexion/extension in the vertical plane, wrist flexion/extension in the horizontal plane with gravity eliminated, wrist radial/ulnar deviation in the vertical plane, wrist radial/ulnar deviation in the horizontal plane with gravity eliminated, finger flexion/extension, and complex movements. In each game, the wearer is required to successfully perform a task that is related to the specific intended movement in order to obtain high scores. The games simulate ADLs, such as catching butterflies or balls, squeezing oranges, fishing, cooking, cleaning the floor, pouring wine, painting fences, and turning over pages, which allows the participants to easily familiarize themselves with the training program and motivates them to perform the tasks.

The intervention in the SG group involved the abovementioned categories of movements of the distal upper extremity in order to achieve goals in a specific task based on visual feedback in real time. In addition, the difficulty of the intervention was adjusted by the artificial intelligence of the system according to participant performance [17]. The function for the algorithm is given by

1 $$ D{L}_i\kern0.75em =D{L}_{i-1}\times \left(1+\alpha \left({P}_{i-1}-{P}_{ref}\right)\right) $$

where *DL*_*i*_ is the difficulty level for the current trial *i*, α is a constant for the rate of update, *P*_*t-1*_ is the performance on the previous trial, and *P*_*ref*_ is the reference performance. In the games, the performance in the algorithm is mostly range of motion for each function movement, but it could be other quantity such as time depending on the games. The reference performance is set to be 80 % of the active range of motion or 80 % of the maximum performance from previous trials. The difficulty level could be position of target, target performance, duration, movement speed, or others depending on the game. The algorithm progressively increases the difficulty level until the current performance is below the reference performance, and it keeps modulating the difficulty level to make the performance stay near the reference performance.

### Conventional intervention

The intervention in the CON group involved the same categories of movements of the distal upper extremity as those in the SG group in order to minimize confounding between the 2 groups. Therefore, all factors, except the use of the SG system, were consistent between the 2 groups. The difficulty of the intervention was adjusted by occupational therapists according to participant performance.

### Outcome measures

The baseline characteristics assessed were age, sex, handedness, time since stroke onset, stroke type, affected body side, and the medical research council scale scores of the flexor/extensor of the shoulder, elbow, and wrist.

#### Primary outcome

Motor impairment of the affected upper limb was evaluated using the upper extremity Fugl–Meyer assessment (FM-total; 33 items with a 3-point ordinal scale; range, 0–66), with higher scores indicating lower impairment [18]. We further divided the FM-total score into proximal (shoulder, elbow, and forearm; FM-prox) and distal (wrist and hand; FM-dist) scores. The primary outcome was the change in the FM scores.

#### Secondary outcomes

Hand function was evaluated using the Jebsen–Taylor hand function test (JTT) and Purdue pegboard test (PPT). The JTT was used to assess hand function mimicking ADLs. It involves a series of 7 timed subtests, including writing, simulated page turning, picking up small objects, simulated feeding, stacking checkers, picking up large light objects, and picking up large heavy objects. Quantification is not possible if a subtest cannot be completed within a certain time, as the result is a continuous time variable, and a subtest is considered to have a missing value if it cannot be completed. Therefore, we used a scoring system (each subtest score ranges from 0 to 15, and the total score calculated as the sum of each subtest score ranges from 0 to 105), which has been shown to have good validity in people with stroke [19]. We used the total score (JTT-total), and divided the JTT-total into gross (stacking checkers, picking up large light objects, and picking up large heavy objects; JTT-gross) and fine hand function (writing, simulated page turning, picking up small objects, and simulated feeding; JTT-fine) scores.

The PPT was used to evaluate fine hand motor proficiency. It involves a board with 2 parallel rows having 25 holes each. The participants are required to pick and place pins into the holes, and the score is the number of pins placed in 30 s. Scores are assessed for the right hand, left hand, and both hands, and the sum of these scores is determined. The test involves 4 trials. Additionally, scores are assessed for the number of assembled pins, washers, and collars in 60 s. We modified the original PPT, and recorded scores for the affected hand (PPT-aff), both hands (PPT-both), and assembly (PPT-assembly).

HRQoL was measured using a stroke-specific, self-reported patient-perspective assessment tool, the Stroke Impact Scale (SIS) version 3.0, which consists of the following 8 domains: strength, hand function, mobility, physical and instrumental activities of daily living (ADLs/IADLs), memory and thinking, communication, emotion, and social participation [20]. The score for each domain ranges from 0 to 100, with higher scores indicating better HRQoL. The hand function, ADLs/IADLs, and social participation scores were combined into a composite SIS score to demonstrate the comprehensive impact of functional change relevant to the interventions used in the present study from the perspective of the international classification of functioning, disability, and health [21]. Additionally, the overall SIS score was calculated as the sum of all domain scores. The secondary outcomes were the changes in the JTT, PPT, and SIS scores.

The FM, JTT, and PPT scores were determined before the intervention (T0), in the middle of the intervention (after the 10th session; T1), immediately after the intervention (T2), and 1 month after the intervention (T3). The SIS scores were determined only at T0 and T2. A trained and blinded outcome assessor who was unaware of group allocation performed all outcome measurements. Adverse events were recorded during the intervention and at outcome measurements.

### Sample size

As this was the first study to assess the efficacy of the SG system in people with stroke, power calculation was performed using FM scores from a previous study, which applied VR-based rehabilitation for the upper extremity in stroke survivors, hypothesizing a similar efficacy between our rehabilitation and the previous rehabilitation [22]. Accordingly, 18 participants were required in each group to provide 80 % power for efficacy evaluation, setting the α level at 0.05. Finally, we calculated that 46 participants were needed, considering a 20 % dropout rate.

### Statistical analysis

An intention-to-treat analysis was performed, which included all participants who were enrolled in the present study regardless of intervention completion. We used the last observed outcome values for the determination of missing values in dropouts, conservatively assuming that no changes occurred after the last observation. At baseline, the mean values of variables were compared between the SG and CON groups using the Mann–Whitney *U* test and Fisher exact test for continuous and categorical variables, respectively. Analysis of variance was performed for repeated measurements in the groups (SG and CON groups) as the between-patient factors and time (T0, T1, T2, and T3) as the within-patient factor in order to compare the effects of each intervention on the FMA, JTT, and PPT scores. The main effects of Group, Time, and Time × Group interactions were evaluated. The Greenhouse-Geisser procedure was applied when the assumption of sphericity was violated, and the post-hoc test was performed. All statistical analyses were performed using SPSS software (version 17.0; IBM, Armonk, NY), and a *P*-value <0.05 was considered statistically significant.

## Results

**Fig. 2:**
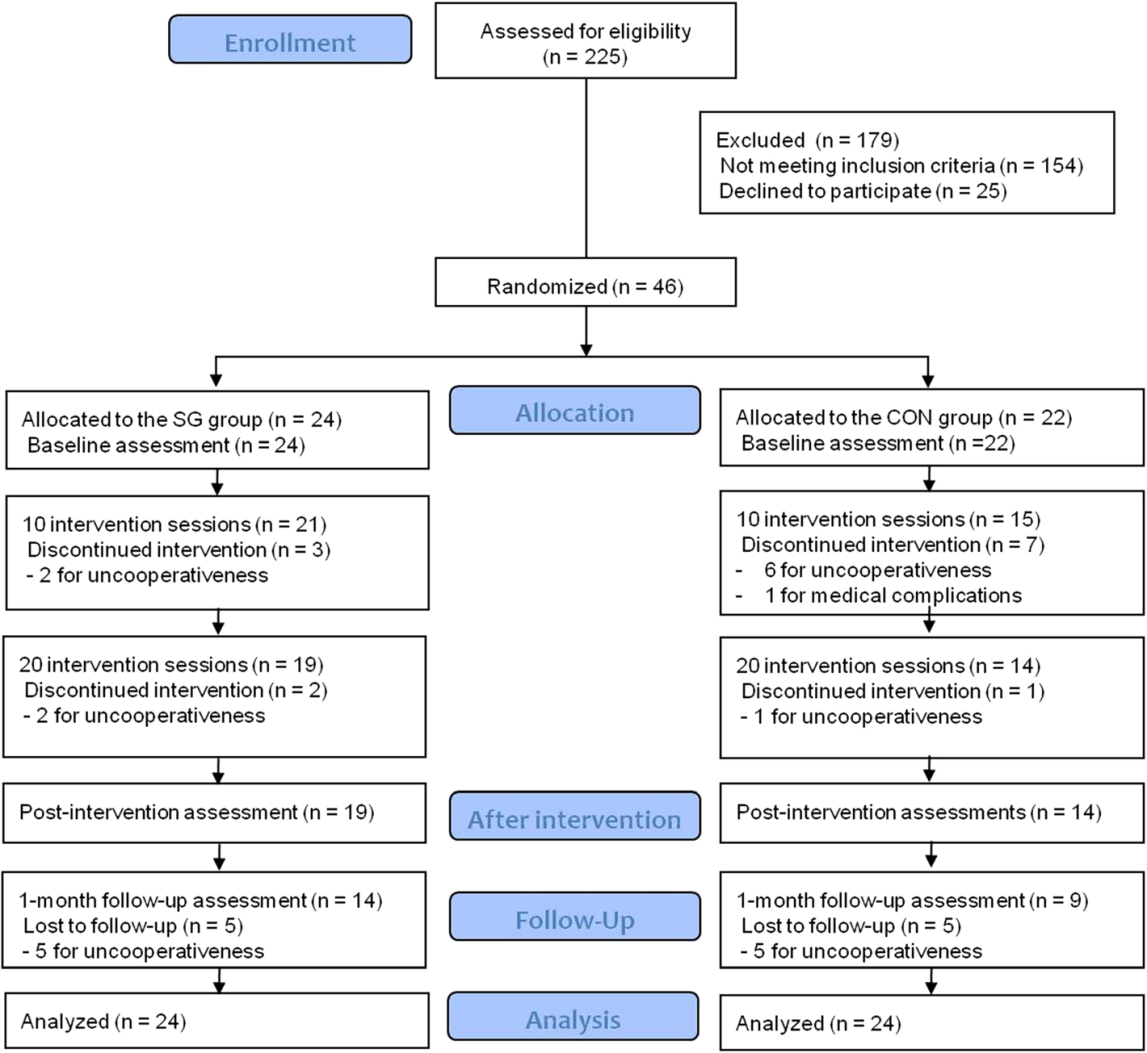
Flowchart of the participants through the study. Abbreviations: SG, Smart Glove; CON, conventional intervention

Of the 46 participants included in the present study, 33 completed the 4-week intervention programs and assessments at T2 and 23 completed the follow-up assessments at T3. During the study, 5 and 8 participants from the SG and CON groups, respectively, did not complete the intervention programs. The sample sizes at the assessment time points are presented in Fig. 2. There were no serious adverse events, and only 1 participant from the CON group dropped out owing to dizziness, which was unrelated to the intervention. Thus, most of the study withdrawals were related to uncooperativeness, and the number was higher than that hypothesized in the study design. At baseline, there were no differences in the demographics and clinical characteristics between the SG and CON groups (Table 1).

**Table 1:** Participant Characteristics

	SG group (*n* = 24)	CON group (*n* = 22)	*P*-value
Demographics			
Age, years	57.2 ± 10.3	59.8 ± 13.0	0.373^a^
Gender, male	19 (79.2)	17 (77.3)	0.578^b^
Dominant hand, right	23 (95.8)	22 (100)	0.522^b^
Stroke characteristics			
Time from stroke, months	13.6 ± 13.4	15.0 ± 14.6	0.809^a^
Affected arm, right	9 (37.5)	11 (50.0)	0.590^b^
Ischemia	15 (62.5)	14 (63.6)	0.590^b^
Clinical characteristics			
MRC scale shoulder flexor	3.2 ± 0.7	3.3 ± 0.7	0.738^a^
MRC scale shoulder extensor	3.3 ± 0.6	3.4 ± 0.6	0.703^a^
MRC scale elbow flexor	3.6 ± 0.6	3.8 ± 0.6	0.472^a^
MRC scale elbow extensor	3.5 ± 0.6	3.7 ± 0.6	0.228^a^
MRC scale wrist flexor	3.4 ± 0.6	3.6 ± 0.7	0.490^a^
MRC scale wrist extensor	3.4 ± 0.7	3.5 ± 0.8	0.719^a^
FM-total score	53.4 ± 8.7	48.2 ± 12.3	0.169^a^

*Abbreviations*: *SG* Smart Glove, *CON* conventional intervention, *MRC* medical research council, *FM* Fugl–Meyer assessment

Values are presented as mean ± standard deviation or number (%). There were no significant differences between groups at baseline for the characteristics

^a^Mann–Whitney *U* test

^b^Fisher’s exact test

### Primary outcomes

**Fig. 3:**
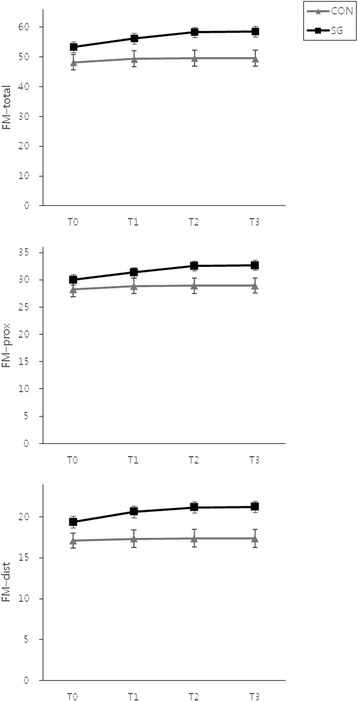
Mean and standard errors for the FM scores in the SG and CON groups. Abbreviations: FM, Fugl–Meyer assessment, SG, Smart Glove; CON, conventional intervention

The FM scores of the SG and CON groups are presented in Table 2. There were no differences in the FM-total, FM-prox, and FM-dist scores between the 2 groups at T0. There were significant improvements in the FM-total, FM-prox, and FM-dist scores in the SG group during the intervention and at the follow-up; however, no significant changes were noted in the CON group (Fig. 3). The improvements in the SG group were supported by significant Time × Group interactions (FM-total: F = 6.48, df = 1.46, *P* = 0.006; FM-prox: F = 5.73, df = 1.705, *P* = 0.007; FM-dist: F = 4.64, df = 1.38, *P* = 0.024).

**Table 2:** FM and JTT Scores in the SG and CON Groups

	SG (*n* = 24)							CON (*n* = 22)						
	T0	T2	Change (T2 − T0)	*P*-value	T3	Change (T3 − T0)	*P*-value	T0	T2	Change (T2 − T0)	*P*-value	T3	Change (T3 − T10)	*P*-value
FM-total	53.4 ± 1.8	58.3 ± 1.7	4.9 ± 1.0	<0.001	58.5 ± 1.7	5.3 ± 1.1	0.001	48.2 ± 2.6	49.6 ± 2.7	1.4 ± 0.8	0.512	49.5 ± 2.7	1.3 ± 0.8	0.592
FM-prox	30.0 ± 1.0	32.5 ± 0.9	2.5 ± 0.6	0.001	32.7 ± 0.9	2.6 ± 0.6	0.001	28.3 ± 1.4	28.9 ± 1.4	0.6 ± 0.4	0.538	29.0 ± 1.4	0.7 ± 0.4	0.471
FM-dist	19.4 ± 0.7	21.2 ± 0.7	1.8 ± 0.5	0.004	21.2 ± 0.7	1.8 ± 0.5	0.007	17.3 ± 1.1	17.4 ± 1.1	0.3 ± 0.5	1.000	17.4 ± 1.1	0.3 ± 0.4	1.000
JTT-total	32.8 ± 5.0	43.1 ± 5.9	10.3 ± 2.7	0.004	43.7 ± 6.1	10.9 ± 2.7	0.003	22.9 ± 5.1	26.4 ± 5.8	3.5 ± 1.4	0.097	26.6 ± 5.9	3.8 ± 1.6	0.152
JTT-gross	14.5 ± 2.4	19.0 ± 2.8	4.5 ± 1.1	0.003	19.3 ± 2.9	4.8 ± 1.2	0.003	10.9 ± 2.5	12.0 ± 2.8	1.2 ± 0.8	0.863	12.7 ± 2.9	1.3 ± 0.9	0.902
JTT-fine	18.3 ± 2.7	24.1 ± 3.2	5.8 ± 1.6	0.008	24.4 ± 3.3	6.2 ± 1.7	0.009	12.0 ± 2.7	14.4 ± 3.1	2.5 ± 1.1	0.158	15.2 ± 3.2	2.7 ± 1.2	0.193

*Abbreviations*: *SG* Smart Glove, *CON* conventional intervention, *FM* Fugl–Meyer assessment, *JTT* Jebsen–Taylor hand function test

Values are presented as mean ± standard deviation

### Secondary outcomes

#### Jebsen–Taylor hand function test

**Fig. 4:**
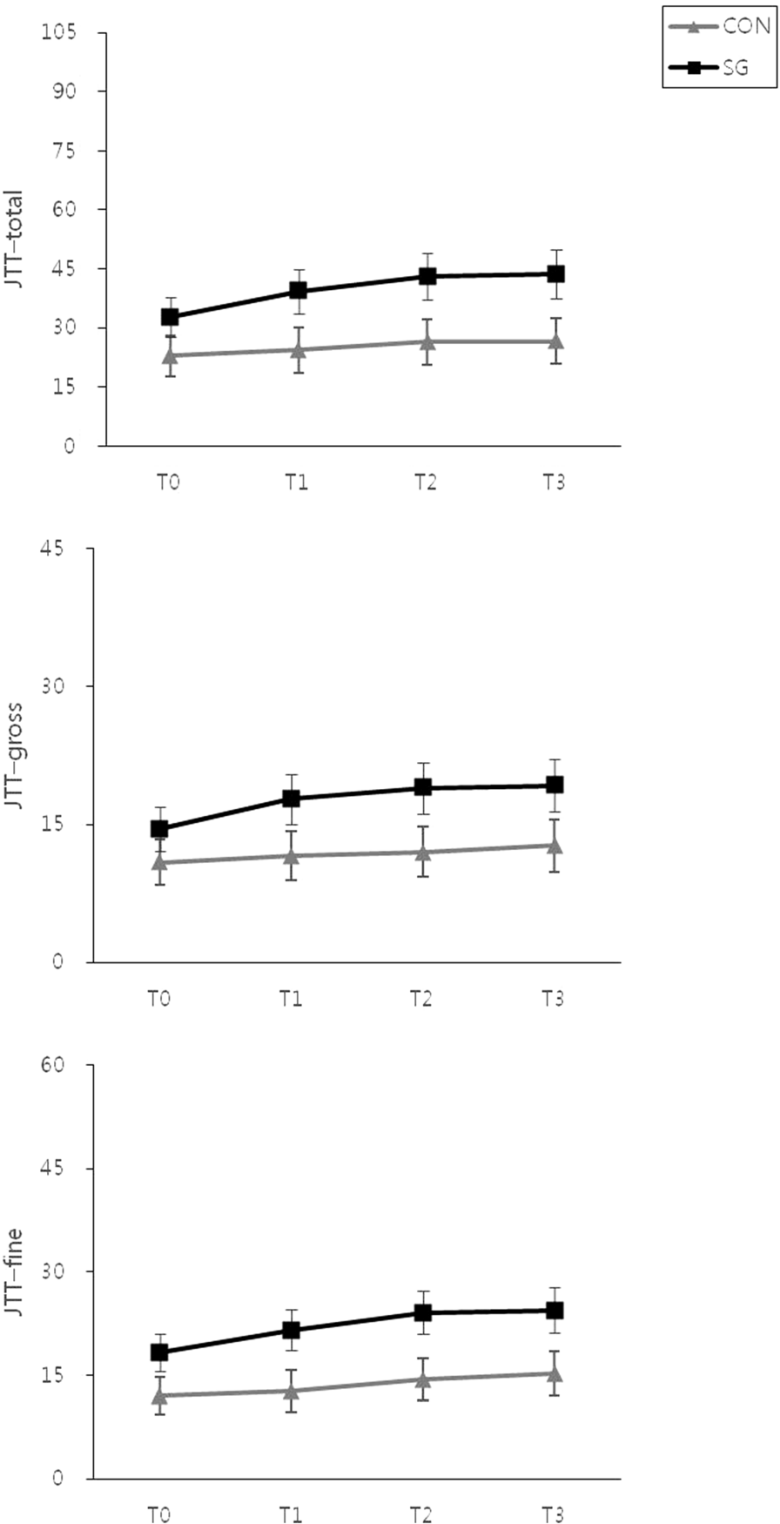
Mean and standard errors for the JTT scores in the SG and CON groups. Abbreviations: JTT, Jebsen–Taylor hand function test; SG, Smart Glove; CON, conventional intervention

The JTT scores of the SG and CON groups are presented in Table 2. There were no significant differences in the JTT-total, JTT-gross, and JTT-fine scores between the 2 groups at T0. The post-hoc test found that there were significant improvements in the JTT-total, JTT-gross, and JTT-fine scores in the SG group during the intervention and at the follow-up; however, no significant changes were noted in the CON group (Fig. 4). The improvements in the JTT-total and JTT-gross scores in the SG group were supported by significant Time × Group interactions (JTT-total: F = 4.073, df = 1.497, *P* = 0.032; JTT-gross: F = 4.155, df = 1.705, *P* = 0.025). However, the Time × Group interaction for the JTT-fine score was not significant, indicating a similar pattern in both groups (F = 2.207, df = 1.493, *P* = 0.131).

#### Perdue pegboard test

The PPT-aff, PPT-both, and PPT-assembly scores were higher in the SG group than in the CON group at T0 (*P* = 0.033, *P* = 0.018, and *P* = 0.009, respectively). The Time × Group interactions for PPT-aff (F = 1.260, df = 1.912, *P* = 0.288), PPT-both (F = 1.016, df = 1.547, *P* = 0.350), and PPT-assembly (F = 1.934, df = 2.265, *P* = 0.288, *P* = 0.144) were not significant, indicating a similar increase in fine hand motor function in both groups.

#### Stroke impact scale

There were no significant differences in the composite, overall, and individual SIS domain scores between the 2 groups at T0 (Table 3). The post-hoc test found that the SG group had significant improvements in the composite (36.7 ± 10.0, *P* = 0.001) and overall SIS scores (61.0 ± 19.6, *P* = 0.005) during the intervention. However, no significant improvements in the composite (1.9 ± 10.5, *P =* 0.856) and overall SIS scores (2.1 ± 15.1, *P =* 0.889) were noted in the CON group. Additionally, the Time × Group interactions were significant for the composite SIS score (F = 5.76, df = 1.0, *P* = 0.021) and the overall SIS score (F = 6.408, df = 1.0, *P* = 0.015). Moreover, among individual domain scores, the Time × Group interactions were significant for the mobility score (F = 5.333, df = 1.0, *P* = 0.026) and the social participation score (F = 5.858, df = 1.0, *P* = 0.020).

**Table 3:** Baseline and Post-intervention SIS Scores in the SG and CON Groups

	T0		T2		Repeated-measures ANOVA	
Outcome variables	SG	CON	SG	CON	F	*P*-value
Strength	33.5 ± 19.9	25.9 ± 4.8	37.0 ± 4.0	29.2 ± 3.9	0.008	0.929
Hand function	45.9 ± 34.2	42.6 ± 6.1	58.5 ± 31.1	42.4 ± 7.0	2.931	0.094
Mobility	68.6 ± 5.4	76.6 ± 5.5	80.0 ± 3.7	75.7 ± 4.6	5.333	0.026
ADLs/IADLs	61.4 ± 18.5	64.2 ± 5.2	74.0 ± 3.3	68.6 ± 4.4	3.534	0.067
Memory and thinking	73.7 ± 4.9	81.1 ± 5.1	73.4 ± 4.0	80.1 ± 5.6	0.167	0.685
Communication	79.4 ± 24.6	84.5 ± 4.5	82.0 ± 24.4	84.2 ± 4.6	2.702	0.108
Emotion	62.4 ± 13.4	64.4 ± 4.1	64.4 ± 16.7	59.4 ± 3.4	3.669	0.062
Social participation	40.4 ± 20.2	46.4 ± 5.8	49.1 ± 21.5	44.2 ± 3.7	5.858	0.020
Composite SIS	147.7 ± 4.1	153.2 ± 14.5	181.6 ± 59.8	155.2 ± 13.3	5.763	0.021
Overall SIS	465.2 ± 121.5	485.8 ± 31.3	518.3 ± 100.2	483.7 ± 28.7	6.408	0.015

*Abbreviations*: *SG* Smart Glove, *CON* conventional intervention, *ADLs/IADLs* activities of daily living/instrumental activities of daily living, *SIS* Stroke Impact Scale

Values are presented as mean ± standard deviation

## Discussion

The present study noted greater improvements in multiple outcomes of the distal upper extremity, including motor impairment (FM-total, FM-prox, and FM-dist scores), hand functions (JTT-total and JTT-gross scores), and HRQoL (composite SIS, overall SIS, SIS-social participation, and SIS-mobility scores) using VR-based rehabilitation with standard OT than using amount-matched conventional rehabilitation, without any adverse events, in stroke survivors. Additionally, this study noted improvements in the SIS-ADLs/IADLs score beyond the minimum clinically important difference (MCID) of 5.9 in the SG group [23]. The improvements in the FMA and JTT scores in the SG group were maintained at the 1-month follow-up.

A previous systematic review found that task-specific training enhanced arm function; however, this review failed to show the beneficial effects of the intervention on hand function [24]. Additionally, a recent systematic review failed to show the beneficial effects of VR-based rehabilitation on distal upper extremity function in stroke survivors [9]. However, our study found that the functional improvements of the distal upper extremity were better using VR-based rehabilitation than using conventional rehabilitation, according to the FMA-dist and JTT-total scores. Furthermore, the functional improvements of the distal upper extremity using VR-based rehabilitation were more definite for gross hand function than for fine hand function, as a significant difference was noted in the JTT-gross score but not in the JTT-fine and PPT scores. Therefore, the improvements in distal upper extremity function using VR-based rehabilitation might have resulted from the task-specificity of the SG system, as the intervention mainly consisted of gross movements of the distal upper extremity without fine movements involving individual fingers. The following are the highlights of task-specific training: relevance to the patient and context, randomly ordered practice sequence, repetition, reconstruction of the task, and positive reinforcement [25]. The SG system includes all the abovementioned properties of task-specific training. The games in the SG system require participants to repeat the reconstructed tasks mimicking ADLs, which are relevant to them and the context. Therapists could prepare specific intervention schedules by combining games; thus, a randomly ordered practice sequence could be prepared. In addition, the artificial intelligence of the SG system can adjust the difficulty of tasks according to participant performance; thus, allowing the completion of the task. This provides a feeling of achievement, which is enhanced by audio or visual feedback in the game, leading to positive reinforcement. Therefore, the task-specific training effects might be maximized using the SG system. A recent study showed that the improvement in distal upper limb function was greater using VR-based rehabilitation with an actuated glove than using conventional rehabilitation, according to the JTT scores [13]. In contrast, the SG system used in our study did not include an actuated apparatus; thus, our results represent the task-specific effects of VR-based rehabilitation without the use of additional tools.

We found that VR-based rehabilitation had beneficial effects on both the proximal and distal upper extremity, which were indicated by the FMA-prox and FMA-dist scores, respectively. These results were not expected, as we believed that the VR-based rehabilitation would only influence the distal upper extremity because the SG system focuses on the distal upper extremity. A possible explanation for the results is that the distal part plays a major role in upper extremity function as an end-effector; therefore, the high activity of the distal part during rehabilitation promoted the active use of the affected upper extremity, which was neglected or not used, thus overcoming learned non-use [3]. Training using the SG system allowed the performance improvement to be generalized to untrained tasks. Recent studies have shown that the effects of VR-based rehabilitation for the upper extremity were transferred to distinct tasks in stroke survivors [10, 13]. Moreover, this extension of performance improvement to untrained tasks after task-specific training was not dependent on the similarity between tasks [26]. Krakauer advocated that a rehabilitation technique should allow the extension of performance improvement to untrained tasks [27]. Therefore, we believe that the SG system is an ideal rehabilitation tool.

We noted greater improvements in the composite SIS, overall SIS, and SIS-social participation, and SIS-mobility scores using VR-based rehabilitation than using conventional rehabilitation. These findings are largely consistent with those of previous randomized controlled trials that showed greater improvements in overall SIS scores or some physical domain scores using constraint-induced movement therapy (CIMT) than conventional therapy [28, 29]. However, a previous study on CIMT reported improvements in only SIS-hand function scores [28]. A Cochrane review on VR commented on the low number of studies regarding participation restriction or QoL [9]. Additionally, a recent systematic review suggested the performance of more studies evaluating the effects of upper limb interventions on HRQoL [30]. A recent study, which was not included in these reviews, showed the possible benefits of VR-based rehabilitation on HRQoL by comparing Short-Form Health Survey scores between VR-based rehabilitation and conventional rehabilitation [31]. One item of the Short-Form Health Survey (role limitation due to physical problem) showed greater improvement after VR-based rehabilitation than after conventional rehabilitation. Our study also showed the beneficial effects of VR-based rehabilitation on HRQoL, including more generalized effects on HRQoL, which are represented by improvements in the overall and composite SIS scores in accordance with our functional results.

The present study had several limitations. First, the improvements in the FM scores did not exceed the MCID of 6.6 points [32]. Additionally, the improvements in the JTT and PPT scores did not have established MCID values. Therefore, it is not appropriate to state that the improvements in the SG group were within the minimum values required to consider the intervention clinically important. However, considering the findings of previous studies on VR (FM MCIDs between 3.5 and 4.5) [9, 33], the improvements in the FM scores were good in the SG group. Therefore, the SG system might be a clinically meaningful VR-based rehabilitation tool. Moreover, the improvement in the SIS-ADLs/IADLs score was beyond the MCID of 5.9 in the SG group. Therefore, the SG system can be considered a clinically useful rehabilitation tool. Furthermore, the ceiling effect of the FM score might hamper the observation of a further improvement in the FM score. Second, the FM score was used as the primary outcome and for power calculation; however, the target of the SG system was the distal upper extremity. We used the FM score, as there was no appropriate VR-based rehabilitation study using outcome measures focused on the hand, such as the JTT. In addition, we hoped to investigate the generalized effects of the SG system on the upper extremity by using the FM score. Future studies using outcome measures relevant to the distal upper extremity are needed to examine the efficacy of the present system. Third, the study did not include a group that received training using only the SG system or a group that received only standard OT. We provided 30 min of standard OT in the SG group from an ethical standpoint because there were no clinical data to support the use of the SG system. Thus, it is difficult to state that the good outcomes in the SG group resulted from the use of the SG system. A future study is warranted to compare rehabilitation using the SG system only with conventional rehabilitation. Fourth, follow-up evaluations were not performed with the SIS; thus, the long-term beneficial effects of rehabilitation using the SG system on HRQoL could not be determined. Fifth, we used a new scoring system for the JTT instead of raw time; thus, comparisons with other results or broader interpretation might be limited. Sixth, participants who exhibited hand flaccidity were excluded, as the SG system does not provide assistive force. We are performing another clinical trial with a combination of functional electrical stimulation and the SG system to overcome this limitation.

## Conclusions

VR-based rehabilitation combined with standard OT might be more effective than amount-matched conventional rehabilitation for improving distal upper extremity function and HRQoL in stroke survivors. Therefore, the SG system used in VR-based rehabilitation might be an ideal rehabilitation tool for the distal upper extremity in stroke survivors.

## Abbreviations

ADLs: activities of daily living
CIMT: constraint-induced movement therapy
CON: conventional intervention
FM: Fugl–Meyer assessment
HRQoL: health-related quality of life
JTT: Jebsen–Taylor hand function test
MCID: minimum clinically important difference
OT: occupational therapy
PPT: Purdue pegboard test
QoL: quality of life
SG: Smart Glove
SIS: Stroke Impact Scale
VR: virtual reality

## References

[1] Kwakkel G, Kollen BJ, van der Grond J, Prevo AJ (2003). Probability of regaining dexterity in the flaccid upper limb: impact of severity of paresis and time since onset in acute stroke. Stroke.

[2] Broeks JG, Lankhorst GJ, Rumping K, Prevo AJ (1999). The long-term outcome of arm function after stroke: results of a follow-up study. Disabil Rehabil.

[3] Wolf SL, Winstein CJ, Miller JP, Taub E, Uswatte G, Morris D, et al. Effect of constraint-induced movement therapy on upper extremity function 3 to 9 months after stroke: the EXCITE randomized clinical trial. JAMA. 2006;296(17):2095–104. 10.1001/jama.296.17.2095 17077374

[4] Fredericks CM, Saladin LK, Fredericks C (1996). Pathophysiology of the motor systems: principles and clinical presentations.

[5] French B, Thomas LH, Leathley MJ, Sutton CJ, McAdam J, Forster A, et al. Repetitive Task Training for Improving Functional Ability After Stroke. Stroke. 2009;40(4):e98–9. 10.1002/14651858.CD006073.pub2 17943883

[6] Liepert J, Bauder H, Miltner WH, Taub E, Weiller C (2000). Treatment-induced cortical reorganization after stroke in humans. Stroke.

[7] Nudo RJ, Wise BM, SiFuentes F, Milliken GW (1996). Neural Substrates for the Effects of Rehabilitative Training on Motor Recovery After Ischemic Infarct. Science.

[8] Maclean N, Pound P, Wolfe C, Rudd A (2000). Qualitative analysis of stroke patients’ motivation for rehabilitation. BMJ.

[9] Laver KE, George S, Thomas S, Deutsch JE, Crotty M. Virtual reality for stroke rehabilitation. Cochrane Database Syst Rev. 2015:CD008349. 10.1002/14651858.CD008349.pub3 PMC6465102 25927099

[10] Merians AS, Fluet GG, Qiu Q, Saleh S, Lafond I, Davidow A, et al. Robotically facilitated virtual rehabilitation of arm transport integrated with finger movement in persons with hemiparesis. J Neuroeng Rehabil. 2011;8(27):0003–8. 10.1186/1743-0003-8-27 PMC3113321 21575185

[11] Tsoupikova D, Stoykov NS, Corrigan M, Thielbar K, Vick R, Li Y, et al. Virtual immersion for post-stroke hand rehabilitation therapy. Ann Biomed Eng. 2015;43(2):467–77. 10.1007/s10439-014-1218-y 25558845

[12] da Silva CM, Bermudez IBS, Duarte E, Verschure PF (2011). Virtual reality based rehabilitation speeds up functional recovery of the upper extremities after stroke: a randomized controlled pilot study in the acute phase of stroke using the rehabilitation gaming system. Restor Neurol Neurosci.

[13] Thielbar KO, Lord TJ, Fischer HC, Lazzaro EC, Barth KC, Stoykov ME, et al. Training finger individuation with a mechatronic-virtual reality system leads to improved fine motor control post-stroke. J Neuroeng Rehabil. 2014;11(1):171. 10.1186/1743-0003-11-171 PMC4292811 25542201

[14] Van Allen MW. Aids to the examination of the peripheral nervous system. Arch Neurol. 1977;34(1):61–1.

[15] Folstein MF, Folstein SE, McHugh PR (1975). “Mini-mental state”: a practical method for grading the cognitive state of patients for the clinician. J Psychiatr Res.

[16] Gagliese L, Weizblit N, Ellis W, Chan VW (2005). The measurement of postoperative pain: a comparison of intensity scales in younger and older surgical patients. Pain.

[17] Choi Y, Gordon J, Park H, Schweighofer N (2011). Feasibility of the adaptive and automatic presentation of tasks (ADAPT) system for rehabilitation of upper extremity function post-stroke. J Neuroeng Rehabil.

[18] Fugl-Meyer AR, Jääskö L, Leyman I, Olsson S, Steglind S (1974). The post-stroke hemiplegic patient. 1. a method for evaluation of physical performance. Scand J Rehabil Med.

[19] Kim JH, Kim IS, Han TR (2007). New scoring system for jebsen hand function test. J Korean Acad Rehabil. Med..

[20] Duncan PW, Bode RK, Lai SM, Perera S, Investigators GAiNA (2003). Rasch analysis of a new stroke-specific outcome scale: the Stroke Impact Scale. Arch Phys Med Rehabil.

[21] Moriello C, Byrne K, Cieza A, Nash C, Stolee P, Mayo N (2008). Mapping the stroke impact scale (SIS-16) to the International Classification of Functioning, Disability and Health. J Rehabil Med.

[22] Piron L, Turolla A, Agostini M, Zucconi C, Cortese F, Zampolini M (2009). Exercises for paretic upper limb after stroke: a combined virtual-reality and telemedicine approach. J Rehabil Med.

[23] Lin K-c, Fu T, Wu C-y, Wang Y-h, Liu J-s, Hsieh C-j, et al. Minimal detectable change and clinically important difference of the Stroke Impact Scale in stroke patients. Neurorehabil Neural Repair. 2010;24(5):486–92. 10.1177/1545968309356295 20053950

[24] French B, Leathley M, Sutton C, McAdam J, Thomas L (2008). A systematic review of repetitive functional task practice with modelling of resource use, costs and effectiveness. Health Technol Assess.

[25] Hubbard IJ, Parsons MW, Neilson C, Carey LM (2009). Task‐specific training: evidence for and translation to clinical practice. Occup Ther Int.

[26] Schaefer SY, Patterson CB, Lang CE. Transfer of Training Between Distinct Motor Tasks After Stroke Implications for Task-Specific Approaches to Upper-Extremity Neurorehabilitation. Neurorehabil Neural Repair. 2013;27(7):602–12. 10.1177/1545968313481279 PMC3769167 23549521

[27] Krakauer JW (2006). Motor learning: its relevance to stroke recovery and neurorehabilitation. Curr Opin Neurol.

[28] Lin KC, Chang YF, Wu CY, Chen YA. Effects of Constraint-Induced Therapy Versus Bilateral Arm Training on Motor Performance, Daily Functions, and Quality of Life in Stroke Survivors. Neurorehabil Neural Repair. 2009;23(5):441-8. 10.1177/1545968308328719 19118130

[29] Wu C-y, Chen C-l, Tsai W-c, Lin K-c, Chou S-h (2007). A randomized controlled trial of modified constraint-induced movement therapy for elderly stroke survivors: changes in motor impairment, daily functioning, and quality of life. Arch Phys Med Rehabil.

[30] Pulman J, Buckley E (2013). Assessing the efficacy of different upper limb hemiparesis interventions on improving health-related quality of life in stroke patients: a systematic review. Top Stroke Rehabil.

[31] Shin JH, Park SB, Jang SH. Effects of game-based virtual reality on health-related quality of life in chronic stroke patients: A randomized, controlled study. Comput Biol Med. 2015;63:92-8. 10.1016/j.compbiomed.2015.03.011 26046499

[32] Gladstone DJ, Danells CJ, Black SE (2002). The Fugl-Meyer assessment of motor recovery after stroke: a critical review of its measurement properties. Neurorehabil Neural Repair.

[33] Turolla A, Dam M, Ventura L, Tonin P, Agostini M, Zucconi C, et al. Virtual reality for the rehabilitation of the upper limb motor function after stroke: a prospective controlled trial. J Neuroeng Rehabil. 2013;10:85. 10.1186/1743-0003-10-85 PMC3734026 23914733

